# Change in Postoperative Weightbearing Protocol Does Not Increase Postoperative Complications Following Hip Arthroscopy for Femoroacetabular Impingement Syndrome

**DOI:** 10.7759/cureus.40859

**Published:** 2023-06-23

**Authors:** Andrea H Johnson, Jane C Brennan, Laura A Stock, Sandra B Levermore, Alyssa Maley, Justin J Turcotte, Benjamin M Petre

**Affiliations:** 1 Orthopedics, Anne Arundel Medical Center, Annapolis, USA; 2 Orthopedic Research, Anne Arundel Medical Center, Annapolis, USA; 3 Orthopedic Surgery, Anne Arundel Medical Center, Annapolis, USA

**Keywords:** postoperative complications, weightbearing restrictions, postoperative rehabilitation, hip arthroscopy, femoro-acetabular impingement

## Abstract

Background

Postoperative rehabilitation protocols, including weightbearing restrictions following hip arthroscopy (HA) for femoracetabular impingement syndrome (FAIS), vary widely among surgeons, from complete non-weightbearing to immediate weightbearing as tolerated; it is unclear if weightbearing restrictions affect short-term outcomes in patients undergoing HA. The purpose of this study is to evaluate patients undergoing hip arthroscopy for FAIS before and after a change in weightbearing protocol, from partial weightbearing with crutches for three weeks to weightbearing and weaning from crutches as tolerated, by examining postoperative outcomes. We hypothesize that the change in weightbearing protocol will have no significant effect on patient outcomes.

Methods

A retrospective review was conducted of 211 patients undergoing hip arthroscopy by a single high-volume surgeon. The change in weightbearing was implemented in February 2022; previously, all patients were toe-touch weightbearing with crutches for the first three weeks postoperatively. Following this change, patients were allowed to weightbear as tolerated with crutches immediately and wean from crutches as tolerated. The patients were divided into two groups: 119 patients pre-implementation (January 2021 to January 2022) and 92 patients post-implementation (February 2022 to December 2022). The primary endpoint was any complication in the first six weeks postoperatively, divided into complications at two and six weeks, emergency department returns in the first 90 days, reoperations in the first 30 days, and pain at six weeks. We also compared patient-reported outcomes at six weeks.

Results

There were no significant differences in demographics between groups. There were no significant differences in postoperative outcomes between patients that had weightbearing restrictions and those that did not when looking at 30 day return to operating room (0 vs 0%, p=1.000), 90-day return to emergency department (8.4 vs. 13.0%, p=0.386), two-week complications (2.5 vs. 6.5%, p=0.279), six-week complications (1.7 vs. 1.1%, p=1.000), pain score at six weeks postoperatively (0.34 vs. 0.33, p=0.971), any pain at six weeks postoperatively 37.8 vs. 32.6%, p=0.523), and six-week Patient-Reported Outcomes Measurement Information System (PROMIS) physical function (PF) score (36.0 vs. 34.5, p=0.330).

Conclusion

Patients undergoing HA after the discontinuation of a mandatory period of protected weightbearing did not experience any significant increase in complications or continued pain, and patient-reported outcomes were similar. Routine postoperative weightbearing restrictions may not be necessary for patients undergoing hip arthroscopy for femoroacetabular impingement syndrome. Further study is required to validate these findings and determine the optimal postoperative protocol for this patient population.

## Introduction

Femoroacetabular impingement syndrome (FAIS) is a relatively recently recognized source of hip pain and has been implicated in the development of osteoarthritis of the hip as well as damage to the acetabular labrum [1,2]. The use of hip arthroscopy for the treatment of FAIS has increased tremendously in the United States in recent years, from 3.6 per 100,000 in 2005 to 16.7 per 100,000 in 2013, and this will likely continue to increase over time [3,4]. There are three types of impingement described in the literature, depending on where the morphologic abnormality lies: cam type, pincer type, or mixed [2]. Cam lesions are commonly found in young adult males and involve a non-spherical femoral head that makes contact with the acetabulum in flexion and reduces femoral head-neck offset [5]. Pincer lesions are more commonly found in middle-aged women and are caused by an over-coverage of the femoral head, as found in coxa profunda or acetabular retroversion [6]. Mixed impingement is a combination of the two morphologies and has features of both.

Rehabilitation protocols, including weightbearing restrictions following hip arthroscopy, vary widely among surgeons, and there isn’t significant evidence to support one protocol over another [7]. Even among high-volume surgeons, standard protocols can vary from immediate weightbearing as tolerated to non-weightbearing for up to six weeks postoperatively [8,9]. There has only been one published study to date that examines patient outcomes before and after modification of the postoperative weightbearing protocol, comparing non-weightbearing to weightbearing as tolerated in a single surgeon’s practice, and finds no difference in patient outcomes between protocols [10]. The purpose of this study is to evaluate patients undergoing hip arthroscopy for FAIS before and after a change in weightbearing protocol, from partial weightbearing with mandatory crutches for three weeks to weightbearing and weaning from crutches as tolerated, by examining postoperative outcomes including complications at two and six weeks, emergency department returns, pain at six weeks, and patient-reported outcomes. We hypothesize that the change in weightbearing protocol will have no significant effect on patient outcomes.

## Materials and methods

Study population and setting

This study was performed at Anne Arundel Medical Center in Annapolis, Maryland, USA. This study was deemed institutional review board-exempt by the institution’s clinical research committee. A retrospective observational study of patients undergoing hip arthroscopy for FAIS was performed to assess whether the shift from a toe-touch weightbearing with crutches for three weeks postoperatively to an immediate weightbearing as tolerated protocol adversely affected patient outcomes. All surgeries were performed by a single surgeon in a single hospital-based outpatient surgery center from January 2021 through December 2022. All patients underwent arthroscopic surgery for a cam or pincer lesion and were surgically treated with bony resection and repair or reconstruction of the labrum and primary repair of the capsulotomy, along with any other necessary procedures including iliotibial band release or lengthening, gluteus medius repair, trochanteric bursectomy, psoas debridement or lengthening, and synovectomy.

Perioperative protocol

All surgeries were performed on an outpatient basis under general anesthesia; regional anesthesia for postoperative pain control was used at the discretion of the surgeon and anesthesiologist. All patients used the same rehabilitation protocols, with the exception of weightbearing restrictions. Prior to February 2022, all patients were toe-touch weightbearing with crutches from weeks zero to three, progressing to weightbearing as tolerated thereafter. Starting February 2022, patients were instructed to weightbear as tolerated with crutches starting postop day zero and wean from crutches as tolerated. Prior to the study period, routine postoperative bracing was discontinued at our institution. Any patient using a postoperative brace was excluded from the analysis. All patients began a supervised rehabilitation program during postoperative week one. Initial range of motion (ROM) restrictions during physical therapy were hip flexion from 0-120 degrees weeks zero to three, abduction from 0-45 degrees weeks zero to three, and no external rotation for two weeks. Strength and ROM with restrictions were progressed per protocol.

Power analysis

An a priori power analysis was conducted to determine that this study had adequate sample sizes to detect medium and large effect sizes for continuous and categorical endpoints, respectively, with 80% power. Cohen’s d was defined as the difference between two means divided by the standard deviation of the data. Cohen described a "d" of 0.20 to be a small effect size, 0.50 to be a medium effect size, and 0.80 to be a large effect size for continuous endpoints. Cohen’s w was defined as a measure of effect size used for chi-squared tests. Cohen described a "w" of 0.10 to be a small effect size, 0.30 to be a medium effect size, and 0.50 to be a large effect size for categorical endpoints. The sample sizes necessary to detect large effect sizes were 52 and 64 for continuous and categorical endpoints, respectively; 128 and 176 for medium effect sizes; and 788 and 1,570 for small effect sizes (Table 1) [11].

**Table 1:** Power analysis: Sample size required to detect different effect sizes Represents total N

Power Analysis	Small (d=0.2)	Medium (d=0.5)	Large (n=0.8)
Continuous Endpoint	788	128	52
Power Analysis	Small (w=0.1)	Medium (w=0.3)	Large (w=0.5)
Categorical Endpoint	1570	176	64

Data collection and analysis

Demographics, surgical details, and complications were manually recorded from the electronic medical record (EMR). The primary endpoint of the study was complications within the first six weeks postoperatively. This was broken down into any complication at two weeks postoperatively, any complication at six weeks postoperatively, and continued pain at six weeks postoperatively. Continued pain at six weeks postoperatively was defined as patient-reported pain at the six-week postoperative visit that was documented in the provider's note. Patient-reported outcomes were collected six weeks postoperatively using the Patient-Reported Outcomes Measurement Information System (PROMIS) physical function score. Univariate statistics (two-sided independent samples t-tests, chi-square tests, and Fisher’s exact tests) were performed to evaluate differences in demographics, comorbidities, and operative characteristics between patients who had weight-bearing restrictions and those that did not. All statistical analyses were performed using R Studio (Version 4.2.2 © 2009-2023 R Studio, PBC). Statistical significance was assessed at p<0.05.

## Results

Two hundred and eleven patients were included in this study: 119 prior to February 2022 who had initial weightbearing restrictions and 92 from February 2022 who had no weightbearing restrictions postoperatively. One hundred thirty-nine (65.9%) patients were female, 148 (70.1%) patients were white, and 189 (89.6%) patients had an American Society of Anesthesiologists (ASA) score less than three, a mean age of 38.9 years old, and a mean body mass index (BMI) of 27.3 kg/m2. Table 2 examines the demographic details between groups.

**Table 2:** Demographics All values are expressed as mean ± SD or n (%); ASA: American Society of Anesthesiologists

Demographic	Weightbearing Restrictions (n=119)	No Weightbearing Restrictions (n=92)	P value
Age	38.1 ± 15.7	39.9 ± 14.0	0.403
Body mass index	26.8 ± 5.11	28.1 ± 5.78	0.112
Sex			0.923
Male	49 (41.2)	61 (66.3)	
Female	77 (64.7)	31 (33.7)	
Non-white race	23 (19.3)	23 (25.0)	0.580
ASA score ≥ 3	13 (10.9)	9 (9.8)	0.967

There were no significant differences between groups in patients that had weightbearing restrictions postoperatively and those that didn’t in age, BMI, sex, race and ASA score.

Table 3 examines differences in postoperative outcomes between patients that had weightbearing restrictions postoperatively and those that did not.

**Table 3:** Postoperative outcomes All values are expressed as mean ± SD or n (%); OR: operating room; ED: emergency department; PROMIS: Patient-Reported Outcomes Measurement Information System

Outcome	Weightbearing Restrictions (n=119)	No Weightbearing Restrictions (n=92)	P-Value
30-day return to OR	0 (0)	0 (0)	1.000
90-day return to ED	10 (8.4)	12 (13.0)	0.386
Two-week complication	3 (2.5)	6 (6.5)	0.279
Six-week complication	2 (1.7)	1 (1.1)	1.000
Pain score within six weeks	0.34 ± 0.47	0.33 ± 0.50	0.971
Any pain within six weeks	45 (37.8)	30 (32.6)	0.523
Six-week PROMIS physical function	36.0 ± 7.29	34.5 ± 7.49	0.330

Twenty-two (10.4%) patients experienced an emergency department (ED) visit in the first 90 days after surgery, although only seven (31.8%) visits were related to surgery and primarily those involved pain or medication issues; the ED returns unrelated to surgery included unrelated medical issues, psychological issues, and traumatic injuries. Twelve (5.7%) patients experienced a complication in the first six weeks following surgery; complications were overall minor, including superficial wound complications (two patients), pain (seven patients), and medication issues (three patients). One patient experienced a deep vein thrombosis, and one patient required postoperative admission for pain control. There were no significant differences between groups when examining 30-day operating room returns, 90-day emergency department (ED) visits, two-week complications, six-week complications, pain score at six weeks postoperatively, any pain reported at six weeks postoperatively, and the six-week PROMIS physical function score.

## Discussion

In this study, there were no significant differences in demographics or postoperative outcomes between patients that had initial weightbearing restrictions and those that were able to weightbear and wean from assistive devices as tolerated. Approximately 10% of patients experienced an ED visit in the first 90 days after surgery, although fewer than one-third of these visits were related to surgery. The most common surgery-related ED visits were for pain or medication issues. Only 5.6% of patients experienced complications within the first six weeks, and most of these were relatively minor, including superficial wound complications, pain, and medication issues. The findings in this study are similar to the findings by Avnieli et al., who found no difference between patients before and after changing the weightbearing protocol after hip arthroscopy, although their study differed in that they moved from non-weightbearing to weightbearing as tolerated, whereas we moved from partial weightbearing to weightbearing as tolerated [10].

Protected weightbearing or non-weightbearing protocols after hip arthroscopy were established for two primary reasons: to protect against hip fracture following osteochondroplasty and to protect the labral and capsular healing [7]. Hip fracture following hip arthroscopy is a rare complication; in a systematic review by Horner et al., only 43 of 31,392 (0.1%) patients studied experienced a postoperative fracture, although early weight-bearing was the largest modifiable risk factor for hip fracture in these patients [12]. The size of the resection of the femoral head-neck junction also plays a significant role in the risk of postoperative femoral neck fracture, with a resection of 10-30% not significantly increasing the risk in biomechanical studies [13]. Protected weightbearing may not be required with smaller resections, but if greater than 30% of the femoral head-neck junction is resected, a period of partial weightbearing is likely needed to protect against fracture [14]. The effect of early weightbearing on the soft tissue repairs is not altogether clear, although a number of biomechanical studies show a limited impact of normal walking motions on the labrum [15-17]. Henak et al. found that the labrum supports only a very small amount of the total load placed on the hip during normal activities, and Safran et al. also found that the average strain on the labrum was very small, with the greatest strain in the anterior labrum noted with flexion and adduction [16,17]. Koh et al. performed a biomechanical study on repaired labrums and found that the suture anchor repair was able to withstand the physiological load of axial weightbearing immediately [15]. While the current study only evaluated short-term outcomes, there were no hip fractures or labral repair failures noted for the duration of the study.

In light of the unclear need for protected weightbearing after hip arthroscopy, the variability in weightbearing protocols is unsurprising. In a study by Cvetanovich et al. that surveyed 31 hip arthroscopy surgeons in North America, most surgeons prescribed a period of protected weightbearing, most commonly partial weightbearing with 20 pounds of foot flat [8]. In a study performed by Rath et al., a survey of international surgeons found a majority allowed immediate weightbearing on labral resection or repair, although there was significant variability noted [9]. A period of protected weightbearing may have benefits in re-establishing normal gait patterns and muscular balance [18-20]. If appropriate gait patterns and normal muscular control aren’t achieved, there can be longer-term consequences for lower extremity function and may increase the risk for new injuries [21,22]. Conversely, the use of crutches and other assistive devices may cause problems in and of themselves. Axillary crutches, the most commonly used assistive device after hip arthroscopy in this study, increase physiologic demand, perceived exertion, and discomfort when compared with other assistive devices [23-25]. A study by Watanabe and Tani found that the use of crutches while walking decreased the accuracy of gait imagery and may increase the risk of falling [26]. The appropriate approach may be the one used in this study: allowing weightbearing as tolerated and weaning from assistive devices as soon as possible, under the direction of physical therapy as needed.

This study does have a number of limitations. Firstly, it is a single institution, single surgeon observational cohort study, and it is possible that our population of patients is not representative of the broader population. This study is also subject to the inherent limitations of a retrospective study, including selection bias and the potential for confounding variables. Secondly, this study had a short follow-up period, which may not be sufficient to identify all possible complications resulting from discontinuing weightbearing restrictions, although one significant complication, a hip fracture, is likely to occur early in the postoperative period [12]. The results of this study also mirror the results of the only other current study on discontinuing weightbearing restrictions after hip arthroscopy, which found no increased complications in the first two years following surgery [10]. Despite these limitations, we feel this study adds a valuable contribution to the literature and may help clarify the appropriate postoperative weightbearing protocol following hip arthroscopy for FAIS.

## Conclusions

Patients undergoing hip arthroscopy after the discontinuation of a mandatory period of protected weightbearing did not experience any significant increase in complications or continued pain, and patient-reported outcomes were similar. Routine postoperative weightbearing restrictions may not be necessary for patients undergoing hip arthroscopy for femoroacetabular impingement syndrome. Further study is required to validate these findings and determine the optimal postoperative rehabilitation protocol for this patient population.
